# VTE Risk assessment – a prognostic Model: BATER Cohort Study of young women

**DOI:** 10.1186/1477-9560-3-5

**Published:** 2005-04-18

**Authors:** Lothar AJ Heinemann, Thai DoMinh, Anita Assmann, Wolfgang Schramm, Rolf Schürmann, Jan Hilpert, Michael Spannagl

**Affiliations:** 1 Centre for Epidemiology & Health Research Berlin, Invalidenstr.115, 10115 Berlin, Germany; 2 Ludwig-Maximillian-University Munich, Klinikum der Universität, Abteilung Haemostasiologe, Ziemssenstr.1, 80336 Muenchen, Germany; 3 Schering AG, SBU Fertility Control/Hormone Therapy, 13342 Berlin, Germany

## Abstract

**Background:**

Community-based cohort studies are not available that evaluated the predictive power of both clinical and genetic risk factors for venous thromboembolism (VTE). There is, however, clinical need to forecast the likelihood of future occurrence of VTE, at least qualitatively, to support decisions about intensity of diagnostic or preventive measures.

**Materials and methods:**

A 10-year observation period of the Bavarian Thromboembolic Risk (BATER) study, a cohort study of 4337 women (18–55 years), was used to develop a predictive model of VTE based on clinical and genetic variables at baseline (1993). The objective was to prepare a probabilistic scheme that discriminates women with virtually no VTE risk from those at higher levels of absolute VTE risk in the foreseeable future. A multivariate analysis determined which variables at baseline were the best predictors of a future VTE event, provided a ranking according to the predictive power, and permitted to design a simple graphic scheme to assess the individual VTE risk using five predictor variables.

**Results:**

Thirty-four new confirmed VTEs occurred during the observation period of over 32,000 women-years (WYs). A model was developed mainly based on clinical information (personal history of previous VTE and family history of VTE, age, BMI) and one composite genetic risk markers (combining Factor V Leiden and Prothrombin G20210A Mutation). Four levels of increasing VTE risk were arbitrarily defined to map the prevalence in the study population: No/low risk of VTE (61.3%), moderate risk (21.1%), high risk (6.0%), very high risk of future VTE (0.9%). In 10.6% of the population the risk assessment was not possible due to lacking VTE cases. The average incidence rates for VTE in these four levels were: 4.1, 12.3, 47.2, and 170.5 per 10^4 ^WYs for no, moderate, high, and very high risk, respectively.

**Conclusion:**

Our prognostic tool – containing clinical information (and if available also genetic data) – seems to be worthwhile testing in medical practice in order to confirm or refute the positive findings of this study. Our cohort study will be continued to include more VTE cases and to increase predictive value of the model.

## Background

Internationally there are several models available to assess the long-term risk of cardiovascular disease and they are broadly used in clinical practice and research [1-3]. A 10-year risk prediction model based on clinical and laboratory data plays an integral part in planning cardiovascular prevention [1]. However, this applies only for the arterial side of the vascular system. We are not aware of any model to predict the long-term risk for venous thromboembolism (VTE) in a similar way.

A prediction of the absolute risk of venous thromboembolism can only be developed on the basis of a specifically designed long-term cohort study. The study reported here is the first long-term, community-based cohorts study that observed genetic and clinical thromboembolic risk factors and their multivariate impact on VTE incidence to address this issue in young women.

A population-based thromboembolic risk factor study started in the mid-1990s in Bavaria, the ***BA***varian ***T***hrombo***E***mbolic ***R***isk study (BATER), focused on women in the reproductive age [4-6]. Clinical and laboratory risk factors for VTE, the lifetime history of relevant conditions or medications, and the family history of cardiovascular diseases were documented from 1993 throughout the follow-up period until 2003, i.e., carefully reviewing complaints or findings possibly related to the occurrence of venous clots.

This study provides a probability scheme that enables identification of women at high risk for VTE compared to women with virtually no risk for a theoretical period of maximal 10 years (defined by the model used). These findings could contribute to weigh the prognostic importance of clinical and laboratory data for medical decisions and better counseling of patients.

## Methods

Material and methods of this long-term cohort study has been described in detail in earlier publications [4-6]. In brief, we used a cohort of 4337 young women (18–55 years) in Bavaria (Germany) who had at least one follow-up.

We collected data from demographics, reproductive life, lifestyle pattern, conditions/diseases, and particularly potential risk factors for VTE through a questionnaire for self-administration. Whenever possible, time-related information was documented. Using this method we were able to set up the common starting point for the cohort as 1993. Telephone enquiries were made to supplement, clarify and verify the data in the questionnaires.

The primary source for the data on VTE was the follow-up questionnaire (self-reported VTE or symptoms potentially compatible with VTE). This information was completed by telephone interviews with the woman and with the treating physician. All available information about diagnostic and therapeutic measures taken was recorded. Clinical data and/or invasive or non-invasive diagnostic procedures were assigned to one of the following categories of likelihood of a VTE:

### Definite VTE

Unequivocal positive finding in at least one imaging test, e.g., phlebography or duplex sonography for deep venous thrombosis (DVT); pulmonary angiogram, VQ scan, spiral computed tomography (spiral CT) for pulmonary embolism (PE).

### Probable VTE

Typical clinical symptoms for VTE without unequivocal imaging test but positive findings in other diagnostic tests (e.g. Doppler US or plethysmography) and subsequent specific therapy over a longer period (low-dose heparin or other anticoagulants).

### Possible VTE

Typical clinical symptoms for VTE and unknown or equivocal result on imaging, only suspicion of VTE suggested by a non-imaging diagnostic tests (such as Doppler US, plethysmography, ECG, blood gas analysis or others for PE) and no subsequent specific therapy (e.g., only short-term low-dose heparin and bandage).

### Potential VTE

Typical clinical symptoms for VTE without further diagnostic tests or negative results or diagnostics unknown. Unspecific therapy – but nonetheless the treating physician maintained the diagnosis VTE based on clinical findings.

All possible and potential "VTE cases" were excluded from the analyses in this paper because of diagnostic uncertainty, i.e. lacking information whether they should be better classified as VTE cases or non-cases.

Women with a history of cancer or with known antiphospolipid syndrome were not in our follow-up study.

#### Laboratory methods

After having given informed consent the women included in this study gave a blood sample at one time point during the observational period (1996/97). An independent ethics committee approved all study related activities.

Whole blood samples were obtained from resting subjects. Blood was put into tubes with trisodium citrate. Plasma was prepared soon after venipuncture by centrifugation for 15 minutes with 3000 to 4000 / min at room temperature and stored at -20°C.

Protein C and antithrombin activities in plasma were measured by chromogenic substrate assays (Dade Behring, Marburg, Germany). For "antithrombin" the activity against factor IIa was determined, Protein C activity was measured after activation of the proenzyme by snake venom. Plasma activities are given as percentage (units/dl) of pooled human normal plasma.

Genomic DNA was isolated by mean of QIAmp^® ^DNA Blood Kit (Qiagen) according to the manufacturer's instructions. The genetic polymorphisms Factor V R506Q (G1691A), the prothrombin promoter G2010A and the 5-, 10-methylenetetrahydrofolate reductase (MTHFR) A223V (C677T) were determined using a multiplex PCR with allele-specific primers slightly modifying a previously described method [7].

All blood tests were performed blinded, i.e. the investigators had no clinical information, and had no access to the clinical database.

#### Method of data analysis

Due to the importance of the temporal relationship the database was structured to accommodate both concurrent as well as time-dependent variables. Concurrent variables are variables, which describe the woman's status at the time of questionnaire response, whereas the outcome variable (VTE) is time-dependent. While concurrent variables were held in a fixed data set, a periodic data set containing information on lifetime exposures and the occurrence of VTE events along a time axis was created for the time-dependent variables of each participant, using months as a unit of measurement. The exposures of interest in this publication, such as VTE risk factors including genetic markers, refer to the baseline time point.

Some of the variables in the database (age, BMI, Protein C, AT) were continuous. These variables were dichotomized in order to define a categorical exposure status (exposed – non-exposed) for the analyses based on incidence or logistic regression. We arbitrarily separated the continuum in two roughly equal intervals such as age under/over 30 or BMI under/over 25 in order to have sufficient case numbers for analyses with further stratification. For protein C and AT we used the 5th percentile (lower 5% of the distribution in non-cases) as cut off point. This limit was considered as usual definition for deficiency and clinically relevant [8].

All analyses concerning the occurrence of VTE events over time were performed by adding up individual observation time (1993 until the last contact) for different exposure-cohorts and in total. The incidence rate of VTE was calculated per 10,000 women years of observation (WY).

The predictive model was developed using the discriminant function analysis technique [9]. This technique permits to determine which of the clinical and laboratory data discriminate best between two groups: future VTE cases vs. non-cases. In other words, this multivariate analysis determines which variables at baseline are the best predictors of a future event.

We multivariately ranked the predictive power of the variables with suspected effect on occurrence of VTE (i.e., possible or established risk factors). Other available information in the database or information not known at baseline (e.g. later occurring conditions like surgery or longhaul flights; see discussion) were not included into the model since they cannot contribute to a predictive model (i.e. the setting for the application of the results of this study is women consulted by a physician independent of an acute VTE event). Technically, we used stepwise discriminant analysis with forward inclusion or backward elimination of variables. The p-value for entry into the model was 0.49 and for removal 0.50.

The p-value of the parameter provided by the discriminant analysis at the last step determines its rank. Variables with the smallest p-value get the highest rank. This permits the comparison of predictive power of potential risk factors – documented at baseline (1993) – for later occurring VTEs.

This technique permits to classify persons by the discriminant function value (separated by the case status). The true incidence of new VTE cases was determined in strata of persons with different pattern of risk profile to characterize groups with lower or higher absolute VTE risk according their risk profile at entry. We used for this analysis those variables that depicted the highest 5 ranks in the stepwise discriminant analysis.

All analyses were performed with the statistical packages SPSS 10.2, SAS 8.2 or STATA 8.2.

## Results

### Description of the cohort

The overall cohort encompasses 4337 women with sufficient information in 1993 and one follow-up at minimum. The observational period for our current analysis was 32,656 WYs since 1993.

The follow-up was continued until 2003 at most, or was otherwise terminated at the time when the last contact was possible to get information about new conditions that may have had occurred. 2076 women could be followed up until 2002/3 (47.9 %), 595 (13.7%) women dropped out between 1999 and 2001, and the largest proportion of women dropped out before 1999 (38.4%). Thus, the follow-up period was censored some time before 2002/3 for approximately half of the cohort members.

Thirty-four new cases of VTE occurred in the observational period. These cases were finally confirmed and categorized according to diagnostic certainty by an independent medical reviewer as definite (n = 31) or probable (n = 3). Cases with possible/potential VTE (n = 17) were excluded from further analyses because of low diagnostic certainty, i.e. it was not clear whether to classify them in the group "cases" or "non-cases".

Out of the 34 definite or probable VTE cases 18 cases (= 52.9%) were associated with "clinical causes for VTE" and 16 (= 47.1%) were so-called "idiopathic" VTEs. The following previously described "acute clinical causes" for VTE the following were observed: 4 with previous VTE, 3 with pregnancy/delivery, 4 after accident, 2 after surgery, 3 with immobilization, and 2 after long travel.

Table 1 depicts the profile of relevant data available at baseline (1993) to get an impression of the group under follow-up.

**Table 1:** Distribution of 10 clinically and 5 genetically relevant variables in a cohort of young women at baseline of the observational period 1993 – 2003. The total number of women in this analysis is 4320, i.e. excluding 17 women with a final diagnosis of a possible/potential VTE. Deviations from this number are due to missing data

Variables			
Continuous variables		n	Mean (SD)
Age (years)		4320	26.0 (8.6)
Life births, number		1910	1.7 (0.8)
BMI^§^		4309	23.3 (4.1)
Protein C (unit/dl)		4315	102.4 (15.8)
AT III (unit/dl)		4316	98.4 (11.3)
			
Categorical parameters			Percent (%)
Own history of VTE	No	4279	99.0
	Yes	41	1.0
Family history	No	3840	88.9
	Yes	480	11.1
Age, alternative	<30	2843	65.8
	≥ 30	1477	34.2
Family history of varicous veins	No	2395	55.4
	Yes	1925	44.6
Family history of MI	No	3830	88.7
	Yes	490	11.3
BMI, alternative	<25	3218	74.7
	≥ 25	1091	25.3
Ever use of hormone replacement	No	4031	93.7
	Yes	270	6.3
Family history of stroke	No	4013	92.9
	Yes	307	7.1
Ever use of oral contraceptives	No	346	8.0
	Yes	3973	92.0
Education level: University entrance diploma	No	3119	73.2
	Yes	1139	26.8
Ever smoker	No	2022	46.8
	Yes	2296	53.2
			
Laboratory parameters			
Factor V Leiden mutation^1^	No	4035	93.7
	Yes	271	6.3
Prothrombin mutation^1^	No	4088	96.6
	Yes	142	3.4
MTHFR^1^	No	1798	42.5
	Yes	2432	57.5
Protein C: ≥ 77(unit/dl)	No	4117	95.4
<77 (5th percentile; unit/dl)	Yes	198	4.6
AT III: ≥ 81(unit/dl)	No	4106	95.1
<81 (5th percentile; unit/dl)	Yes	210	4.9

^1 ^Homozygote & heterozygote together

^§ ^Body mass index (kg/m^2^)

The mean age was 26 ± 8.6 years, however, for the dichotomized age variable we used as cut-off point 30 years resulting in strata that contained VTE cases in both age groups. The frequency of other conditions, family history of potentially relevant diseases, lifestyle pattern and genetic tests is provided in the table 1. Homo-and heterozygote carriers of mutations were analyzed together because of small numbers of homozygote carriers.

### Ranking risk factors according predictive power for VTE

The stepwise discriminant analysis was used to rank relevant variables at baseline according to their power to predict the case and non-case status many years later. We initially used 7 clinically available, potential risk factors for VTE and 5 laboratory parameters. Later, we combined genetic markers (Factor V Leiden and Prothrombin mutation G20210A) together in one composite variable due to low prevalence in the cases: FVL (n = 4) and PTM (n = 2). We used only categorical variables in the model, i.e., dichotomized continuous variables.

We got the following ranking of the predictive power to explain the occurrence of new VTE cases during a theoretical 10-year period (defined by our model) in declining order: Medical history of VTE, family history of VTE (1^st ^degree relatives), age at baseline 1993, body mass index (BMI), factor V Leiden (FVL) or prothrombin mutation (PTM), family history of varicose veins, protein C, AT level, hormone ever use, MTHFR carrier status, and OC ever use. We considered only the 5 highest-ranking variables for the development of our predictive model for practical use (Table 2). Seven other variables with lower predictive importance were left out because their multivariate impact was too low and the practical application of a model with more than 5 variables is not easy to handle in practice. In addition, models that included other or more than the selected 5 variables brought no further improvement of the prediction (data not shown).

**Table 2:** Ranking order of clinical and laboratory data according, possibly relevant for VTE. Analysis with stepwise discriminant analysis

**Rank order**	**P value**	**Independent variables**
1	0.000	*Medical history of VTE (yes, no)*
2	0.005	*Family history of VTE (yes, no)*
3	0.012	*Age at baseline 1993: <30 vs. ≥ 30 years*
4	0.082	*BMI: <25 vs. ≥ 25*
5	0.112	*FVL or PTM carrier: any positive vs. all negativ*
6	0.153	*Family history of varicose veins (yes, no)*
7	0.536	*Protein C: <77 vs. ≥ 77(unit/dl)*
8	0.767	*AT III: <81 vs. ≥ 81(unit/dl)*
9	0.773	*HRT ever use (yes, no)*
10	0.776	*MTHFR carrier (yes, no)*
11	0.798	*OC ever use (yes, no)*

Taking the VTE incidence in all combinations of the five variables into account, we formed four levels of future VTE risk (Table 3). Some of the combinations of the five risk markers had not sufficient data, i.e. the observation period (WY) was too short to observe new VTE cases.

**Table 3:** Expected risk level for VTE within next 10 years in four categories in the BATER study population: No/low risk, moderate risk, high risk, very high risk.

**Future risk level**	**Study population**		**WY^1^**	**VTE cases**	**VTE incidence**
	**N**	**%**	**years**	**N**	**Per 10^4^WYs**
No/low	2634	61.3	19282	8	4.1
Moderate	907	21.1	7314	9	12.3
High	257	6.0	2117	10	47.2
Very high	40	0.9	352	6	170.5
No data^2^	457	10.6	3300	0	0.0

^1^WY = representing the women-years of observation in the respective category of the BATER cohort)

^2^No cases observed in these sub-groups

The majority of the study population (61.3%) had a small VTE risk (no/low in Table 3), about 20% depicted a "moderate risk", 6% a "high risk", and 0.9% a "very high risk". About 10% could not be classified due to lacking VTE cases in the observation period ("no data"). The cut points for the four risk levels were arbitrarily defined: The average VTE incidence per 10^4 ^WYs steeply increases across the five groups: 4.1 (no/low), 12.3 (moderate), 47.2 (high), and 170.5 (very high).

**Figure 1:**
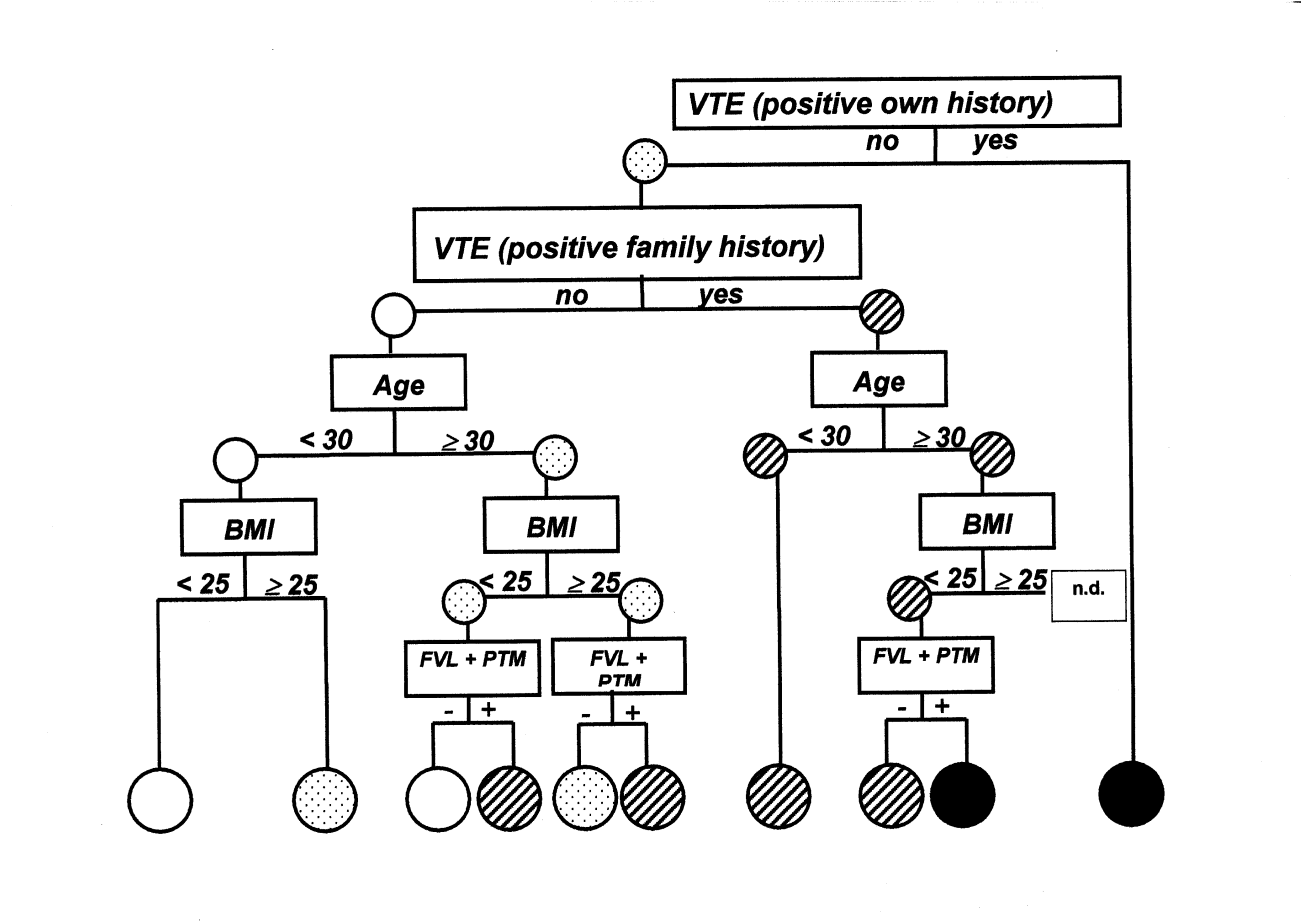
Predicted risk for VTE within next 10 years in four categories: No/low risk (blank circles), moderate risk (dotted), high risk (hatched), very high risk (black circles). BMI = body mass index; FVL + PTM = Factor V Leiden mutation (hetero-&homozygote) and /or prothrombin G20210A mutation (hetero-&homozygote) ; n.d. = no data

Figure 1 provides a scheme to support the individual decision about a future VTE risk based on information on five or less variables. The small number of new VTE cases in our model, however, prevented us from drawing a complete decision tree, i.e. some branches of the tree cases were not observed yet or only one case (considered as not sufficient to be included in the scheme). For example, the small group of VTE cases with a previous history of a VTE in our cohort (n = 4) did not allow for further specification by age, family history of VTE, BMI, and genetic marker: this group is associated with a very high risk altogether, but it is not clear if subgroups may have a lower or higher risk. Other examples were women without previous VTE history but positive family history of VTE, higher age, and higher BMI: there were no cases to distinguish between carriers of genetic markers (FVL or PTM positive) and those without any positive genetic markers.

Women without VTE history, no family history, young and slim are privileged with low future VTE risk. With increasing age and BMI the VTE risk increases, particularly if associated with positive family history and markers for inherited VTE risk. Using this scheme, it is also possible to predict future VTE risk without knowledge about genetic risk factors and to give appropriate advice.

## Discussion

To our knowledge, long-term, community-based cohort studies with the aim to evaluate or compare the predictive power of clinical as well as genetic risk markers for VTE are lacking. Most studies related to VTE risk factors were restricted to clinically available markers such as age, BMI, previous VTE, family history, or acute factors (immobilization, surgery, accidents, pregnancy, and also hormone use) and usually based on clinical observations or case-control studies or studies in administrative databases, and also a few cohort studies (overview about incidence and risk factor studies in [10,11]). One recent publication of a historic cohort [12] assessed carefully the impact of clinical risk factors for the prevalence of VTE and determined relative VTE risk estimates. Cohort studies in the population rarely included/reported genetic markers for thrombophilia and acquired risk factors, except the Physicians Health Study for example – however only for males over 40 years of age [13]. An important recently published cohort study in Denmark analyzed specifically the incidence of VTE and compared carriers of FVL compared with non-carriers [14].

Other studies with focus on markers for hereditary thrombophilia were performed in patients (e.g. in anticoagulation clinics), in relatives of carriers of genetic mutations but not in the "normal female population" [15-18]. In addition, the evaluation of the importance of genetic markers for VTE risk does rarely consider the impact of clinically available risk factors and the design is often restricted to case-control studies. Overall different study designs and restricted views may come to similar conclusions in an ideal world, but not necessarily.

Our BATER cohort study covers more than 4,000 cohort members with a fairly long observation period (1993 – 2003), translating into over 32,000 WYs. Thirty-four VTE cases, classified as definite or probable, occurred within this period. This is equivalent to an incidence of about 10 per 10,000 WYs. It is important to realize that we put great effort on the detection of potential cases and – even more important – we included all definite and probable cases, whereas most reported incidence rates refer only to definite and so-called "idiopathic VTE", i.e. most reported rates excluded all cases that occurred in temporal relationship to possible "acute" causes such as pregnancy/delivery, surgery, and immobilization. Idiopathic VTEs, however, reflect only a part of all confirmed VTE cases [17]. We found in our cohort study roughly 50% so-called "idiopathic" VTE cases, and the other 50% of cases had a previous VTE in the past, pregnancy, delivery, surgery, accident, or immobilization/long bed-rest shortly prior to the VTE event. Thus, the incidence of "idiopathic VTE" observed in this study would be 5 per 10,000 WYs and thereby likely to be in the same range as other reported incidence rates in the general population. The incidence estimates for definite VTE ranges between 1 to 6 per 10^4 ^WYs in OC non-users and 2 to 10 per 10^4 ^WYs in OC users [10]. Older studies depicted almost always-higher incidence rates than more recently performed studies (see overview in [10]). A recent systematic review [11] came to a pooled incidence of definite VTE for the general population of 5 per 10,000 person years, similar in males and females, and found that around 40% of VTE cases were "idiopathic". A large cohort study found a similar incidence rate in males aged 40–49 years: 4.7/10^4 ^person-years [13].

The objective of this study was to provide a simple algorithm for medical practice to predict the future VTE risk with a simple scheme based on usually available information, i.e. to discriminate women with virtually no VTE risk in the foreseeable future from those at a high absolute risk to suffer from VTE. Incidence rates associated with different clinical and genetic factors will be published separately [6].

It is a limitation of this long-term cohort study, however, that the number of confirmed (definitive and probable), incident VTE cases was still too small in absolute numbers (n = 34). In other words, some sub-cohorts with certain combinations of risk factors did not contain one single new VTE case. The consequence was that the number of subgroups at risk was minimized to the extent possible to make it a feasible tool for the practice. In so far, the results and conclusions should be considered as rough but the best we can possibly do at this stage, i.e. future analyses will benefit from an improved point of departure (more cases, longer observation). Another limitation is that we did not have the chance yet to test the validity of the model in another, independent cohort. This is a task for the future. Therefore we focused this paper on a simple scheme with a rough classification of the future VTE risk.

The interested (or worried) women and her treating physician might like to know (or to get confirmation) whether the future VTE risk is higher than "normal" (no/low risk). This information could have an impact on further medical surveillance, especial counseling, proposals as how to reduce of changeable risk factors or on suggestions for preventive measures under certain circumstances and – of course- with respect to the compliance regarding preventive measures.

Using stepwise discriminant analysis the rank order of 12 (11) clinical or laboratory data at baseline (1993) was multivariately determined concerning the power to predict future VTEs. This was the information needed to select a minimal set of parameter combinations to build a "VTE prediction model". Finally we ended up with a model covering the five variables with highest ranking (impact) regarding predictive importance for future VTE only: history of previous VTE, family history of VTE, higher age, higher body mass index, and carrier of FVL or PTM. The decision to form the composite genetic marker "FVL or PTM" was guided by the small numbers of cases who were carrier of this two mutations and the low predictive power of all other lab parameters we had in the data set (see table 2).

Four levels of future VTE risk were arbitrarily defined-based on a steeply increasing absolute VTE risk: *No/low risk *(4 per 10^4 ^WY), *moderate *(12/10^4 ^WY), *high *risk (47/10^4 ^WY), or *very high *(171/10^4 ^WY). The low-risk group was chosen to reflect an assumed VTE risk of the normal population (see above), the group with "moderate risk" because VTE risk over 10/10^4 ^is indicative for an increased risk, and the "high and very high risk" groups are clearly out of the normal range. One should also consider in this context, that these cut-off points reflect an average risk with an assumed variation within these groups – as can be seen in the scheme of a decision tree (figure 1).

In accordance with clinical experience the overwhelming majority (61%) depicts a low risk of a future VTE. Only a minority of 6% and 0.9% is facing a high or very high VTE risk. The women who fall into the two highest risk categories have a previous own history of VTE or a positive VTE family history, have a higher BMI or a genetic mutation (FVL or PTM). Even though, the contribution of genetic appears to be limited. Using an analysis based only on clinically available data, i.e., without use of the information about lab parameters, we arrived at very similar three risk categories with almost identical absolute VTE risk (data not shown separately but are part of Figure 1).

Another point for discussion is the impression suggested by figure 1 that persons with previous VTE do not require genetic testing because they are in the "high risk" category without any further considerations. The risk might well be different for persons with/without inherited risk (family history), younger/higher age, or overweight. This however we cannot further disentangle because we are lacking new VTE events particularly in this high-risk group of our study. Thus, the conclusions are rather crude as discussed before and require clinical experience for the interpretation of individual cases. The need for genetic testing depends on the judgment in a specific clinical situation and the usefulness of this additional information for the physician and/or the patient (family).

Decisions based on clinical variables about preventive measures will be made in any case – even if no genetic information is available. The possible approaches are a matter of a current controversy in the literature [15,20,21]. Clinical reports point often towards a high VTE recurrence rate in patients with previous VTE [22], but despite being the "best" single predictor we found this phenomenon only in 4 of our 34 incident VTE cases.

The predictor variables used in our model seem to be plausible and consistent with the clinical experience: History of previous VTE, age and obesity are indeed important clinical information for the VTE risk assessment, and also genetic marker were discussed as predictors of a future VTE. These are also risk parameter that are commonly used when recommending preventive measures in situations like long-haul flights, immobilization (such as accidents, surgery) and are also labelled as risk factor in drugs containing sexual hormones (e.g., oral contraceptives or hormone therapy).

We assume that physicians will appreciate these results as a possibility to double-check if their own decision are supported by evidence coming from this large cohort study or may even alter their decision. At least the proposed model contains a reassuring element. It should be underlined that these algorithms do not obviate the need of weighing individual risks and benefits.

We conclude from our observation that the prediction of future VTEs can well be done on clinical data alone – at least until better genetic markers are established. In other words, well-established genetic parameters alone are relatively weak long-term risk factors, the occurrence of VTE requires interaction of both inherited and acquired risk factors [23]. Results of several recent studies support arguments against the possibility that testing for thrombophilia could help to better predict future VTEs [15,20,21]. Nonetheless one can argue that genetic testing in families with significant family history of VTE or previous experience of a VTE might well give additional information for clinical decisions and may increase efforts to comply with preventive measures. The limitation of our study is that we cannot further divide the risk spectrum in these sub-groups due to small numbers of new events or too short total WY of observation. In any case, when a genetic test is recommended, the physician should know how a positive test would influence his/her clinical judgment [15]. These early results of our cohort study contribute to this interpretation or decision-making, respectively.

Forecast of VTE risk cannot be based on genetic characteristics alone but only in combination with important clinical data (acquired risk information). Genetic markers play obviously a limited role in the long-term prediction of VTE – at least in the age group under 50. Genetic markers together with these "personal characteristics" constitute the disposition. Family history of cardiovascular events, specifically venous events have to be taken into account. As described before and confirmed by our data the probability whether the disposition translates into an event is obviously more influenced by "personal characteristics" such as higher age, or higher BMI. However, there are obviously other important, more acutely affecting environmental factors such as immobilization, surgery, accidents, and treatment with drugs that influence coagulation. The latter factors can be used to reduce the risk as estimated by the model (or own clinical experience).

Another issue for discussion is the validity of our calculated incidence rates: The lowest VTE incidence level observed in this model was 4 per 10,000 WYs. Due to an active search for findings compatible with the diagnosis of VTE, the inclusion of definite and probable diagnosis as well as of so-called "non-idiopathic VTEs", the incidence figures were expected to be higher than in normal "medical statistics" or administrative databases as discussed above. The equivalent incidence rate for only definite and idiopathic VTE could be expected to be approximately 2 per 10^4 ^per year and therefore very low for women who were using oral contraceptives as the average population. We like to stress that our study was rigorous in documenting the VTE diagnosis. We conclude that the data can be generalized for the female population in the fertile age range. In analogy, the incidence might be compared with results of a prospective, community-based cohort study [13] that found in males aged 40–49 years a VTE incidence rate of 2.7 primary VTEs cases per 10^4 ^person-years (equal to idiopathic: no previous VTE history, no cancer, no surgery or trauma).

The influence of other, more acutely acting risk modifiers – such as immobilization, surgery, long-haul flights, and use of drugs (e.g. OCs and other hormones) was intentionally excluded from this model. Only parameters that were available at baseline and likely to affect the long-term development were eligible for this prognostic model. We saw no possibility to introduce parameters in the model that may or may not operate later, shorter or longer during the observational period. In other words, only long-term characteristics at baseline (both clinical and genetic variables) were initially included into the modeling. Other influential risk factors or preventive measures have to be considered when discussing activities to reduce a predicted increased risk in the medical practice. It was not the aim of the study and data are neither available to test the effect of preventive measures nor the effect of additional risk factors in the immediate period before the event occurred. This would require another study design and a separate study with sufficient power for such questions.

The variables selected for the model fit the expectations of the skilled clinician. The model was developed to assist physicians- we hope for feedback from medical practice.

This "prognostic model" seems to be worthwhile to be tested in clinical practice. There is a minority of women that would need additional genetic testing, intense counseling, suggestions for risk reduction (if possible), and efforts to prevent avoidable risk situations (e.g. treatment with OCs or hormones) or to take other appropriate preventive measures in situation of an acute risk (immobilization, surgery, long-haul flights and others), e.g. compression stockings/ heparin. Several other options to reduce the VTE risk profile are principally available, but sometimes not easy to achieve (e.g. reduction of BMI). Prevention of VTE in medical practice can be improved if the main risk factors are known (importance to document medical history and established risk factors), but also knowledge of their relative importance in the risk-network as well as of interactions with environmental factors. The predictive models discussed in this paper may assist doctors to pay particular attention to labeling prior to prescription (e.g. oral contraceptives or hormones) and to use convincing evidence-based data when counseling women of a predicted higher risk.

We abstained – at the current stage – from the temptation to use a complex equation to calculate an apparently exact risk for the individual person (e.g., using a "risk calculator") because it suggests inadequate accuracy and we rather prefer to provide a very simple scheme that can be handled during routine work. Moreover, we are planning a validation of this model in an independent cohort study, as the first step the part related to clinical risk factors.

## Conclusion

Our prognostic tool – containing clinical information (and if available also genetic data) – seems to be worthwhile testing in medical practice in order to confirm or refute the positive findings of this study. Our cohort study will be continued to include more VTE cases and to increase predictive value of the model.

## Competing interests

The five authors from research institutes (LAJH, TDM, AA, WS, MS) and the two authors from industry (RS, JH) see no conflict of interest.

## Authors' contributions

LAJH: designed together with WS the cohort study and both are the principal investigators, LAJH planned all analyses, wrote a first draft of the manuscript. DMT: developed and maintained the database, performed the majority of analysis, and contributed to the manuscript. AA: responsible for running all field work, performing quality control and designing the validation of diagnoses, contributed to the manuscript. WS: one of the PIs, contributed to the manuscript. RS: responsible together with JH for interpretation of the findings, major contributions to the manuscript. JH see RS. MS: responsible for the haemostasiological lab work during the entire study period, contributed to the manuscript.
